# The impacts of migraine and anxiety disorders on painful physical symptoms among patients with major depressive disorder

**DOI:** 10.1186/1129-2377-15-73

**Published:** 2014-11-10

**Authors:** Ching-I Hung, Chia-Yih Liu, Ching-Yen Chen, Ching-Hui Yang, Shuu-Jiun Wang

**Affiliations:** 1 Department of Psychiatry, Chang-Gung Memorial Hospital at Linkou and Chang-Gung University College of Medicine, Tao-Yuan, Taiwan; 2 Department of Nursing, Chang Gung University of Science and Technology, Tao-Yuan, Taiwan; 3 Department of Neurology, National Yang-Ming University School of Medicine and Taipei Veterans General Hospital, No. 201 Shi-Pai Road, Section 2, Taipei 112, Taiwan

**Keywords:** Depression, Anxiety, Headache, Pain, Somatization, Prognosis

## Abstract

**Background:**

No study has simultaneously investigated the impacts of migraine and anxiety disorders on painful physical symptoms (PPS) among patients with major depressive disorder (MDD). The study aimed to investigate this issue.

**Methods:**

This open-label study enrolled 155 outpatients with MDD, who were then treated with venlafaxine 75 mg per day for four weeks. Eighty-five participants with good compliance completed the treatment. Migraine was diagnosed according to the *International Classification of Headache Disorders*. MDD and anxiety disorders were diagnosed using the Structured Clinical Interview for DSM-IV-TR. The visual analog scale (VAS) was used to evaluate the severity of eight PPS. Multiple linear and logistic regressions were used to investigate the impacts of migraine and anxiety disorders on PPS.

**Results:**

Compared with patients without migraine, patients with migraine had a greater severity of PPS at baseline and post-treatment. After controlling for demographic variables and depressive severity, migraine independently predicted the intensities of eight PPS at baseline and four PPS post-treatment. Moreover, migraine independently predicted poorer treatment responses of chest pain and full remission of pains in the head, chest, neck and/or shoulder. Anxiety disorders predicted less full remission of pains in the abdomen and limbs.

**Conclusion:**

Migraine and anxiety disorders have negative impacts on PPS among patients with MDD. Integrating the treatment of migraine and anxiety disorders into the management of depression might help to improve PPS and the prognosis of MDD.

## Background

Depression and painful physical symptoms (PPS) are closely related and interact [1,2]. PPS are common among patients with major depressive disorder (MDD) [1-5]. MDD patients with PPS have a greater severity of depression [3,5], a poorer quality of life [6,7], and an increased suicidal risk [8]. Moreover, PPS also has negative impacts on the treatment response of depression [9,10].

Migraine is common among patients with MDD and is related to other PPS [11-14]. MDD patients with migraine are associated with greater severities of depression and anxiety and a poorer health-related quality of life [11,13,15]. Migraine is an independent factor predicting somatic symptoms among patients with MDD [16]. Moreover, migraine also has negative impacts on the recovery of health-related quality of life among MDD patients [15].

Patients with depression are often comorbid with anxiety, which is related to a greater severity and a poorer treatment prognosis [17,18]. Anxiety disorders also interact with PPS [19-21]; for example, a patient with generalized anxiety disorder (GAD) has a greater severity of PPS as compared with a control [21].

As described above, MDD, migraine, anxiety disorders, and PPS are closely related and interact [1,2,14,21,22]. Although many studies have investigated the impacts of migraine and anxiety disorders on patients with MDD [12,16-18,23], no study has reported the impacts of migraine and anxiety disorders on PPS among patients with MDD, to the best of our knowledge. Investigating the above issue is mandatory. If migraine and anxiety disorders are important factors related to PPS among patients with MDD, treatment of migraine and anxiety disorders might directly or indirectly improve PPS. There is a possibility that improvement of PPS might be helpful for improving the prognosis of depression, because PPS have a significantly negative impact on the prognosis of patients with MDD [3,5-10]. Therefore, the purpose of this study was to investigate the impacts of migraine and anxiety disorders on PPS among patients with MDD. We hypothesized that migraine and anxiety disorders have negative impacts on PPS among patients with MDD.

## Methods

### Subject enrollment

This project was conducted from September 2005 to August 2007 in the psychiatric outpatient clinics of Chang Gung Memorial Hospital, a medical center in northern Taiwan. The project was approved by the Institutional Review Board of the same hospital. Study participants were recruited from consecutive outpatients aged 18–65 years who had not taken antidepressants or other psychotropic drugs within the previous four weeks. MDD and anxiety disorders were diagnosed using the Structured Clinical Interview for DSM-IV-text revision (TR) Axis I Disorders [24]. Patients who met the DSM-IV-TR criteria for MDD and were experiencing a current major depressive episode were considered eligible subjects [25]. To prevent depression and somatic symptoms from being confounded by other medical conditions, substance abuse, or psychotic symptoms, the following exclusion criteria were established: 1) a history of substance dependence or abuse without full remission in the previous month; 2) psychotic symptoms, catatonic features, or severe psychomotor retardation with obvious difficulty in being interviewed; and 3) chronic medical diseases such as hypertension, diabetes mellitus, and other medical diseases, except for headaches. Written informed consent, based on the guidelines regulated in the Declaration of Helsinki, was obtained from all subjects prior to study enrollment following an in-depth explanation of the procedures.

The study was designed as an open-label study and the eligible subjects were informed about the following issues: 1) Antidepressants used in the study have been proven for the treatment of depression; 2) The period of observation with a fixed-dosage antidepressant (venlafaxine extended-release, one 75 mg capsule per day) was four weeks; 3) Subjects were educated regarding depression and treatment issues. An information sheet was provided to ensure that all subjects received the same information.

### Assessment of headaches

All patients completed a structured headache intake form, which was designed to meet the operational criteria of the *International Classification of Headache Disorders*, 2nd edition (ICHD-2) [26]. Questions regarding headache frequency, intensity, pattern, duration, location, aura, aggravation by physical activities, nausea, vomiting, photophobia, phonophobia, precipitating factors and painkiller use were included. An experienced headache specialist (SJW), blind to the psychiatric evaluation results, interviewed all patients after they had completed the headache intake form and made headache diagnoses. Then, the headache diagnoses were updated to ICHD-3 beta [27]. Subjects who fulfilled the criteria of migraine without aura and/or migraine with aura were categorized as the “migraine” group, while the other patients were classified as the “non-migraine” group.

### Assessment of anxiety disorders

One psychiatrist, blind to the diagnoses of headaches and the psychometric assessment results, used the Structured Clinical Interview to diagnose the following anxiety comorbidities: panic disorder, agoraphobia, social phobia, specific phobia, post-traumatic stress disorder, obsessive–compulsive disorder, and GAD [24]. Subjects with any one of the anxiety comorbidities in a current episode or partial remission were categorized as the “anxiety disorders” group, while the others were categorized as the “non-anxiety disorders” group.

### Instruments

The Hamilton Depression Rating Scale (HAMD) was used to evaluate the severity of depression [28]. Subjects evaluated their average pain intensity during the previous week using a visual analog scale (VAS), with 0 representing “no pain” and 10 representing “pain as severe as I can imagine.” The number of days suffered from each pain in the past week was recorded. Eight pains were evaluated, including pain in bone and/or joints (bone/joints), head, back, abdomen, chest, neck and/or shoulder (neck/shoulder), general muscle, and limbs. The improvement percentage (IP) of pain intensity was calculated by (VAS scores at baseline – VAS scores at the follow-up) × 100% / VAS scores at baseline. An IP of pain intensity ≥50 following the four-week treatment period was defined as “treatment response” and an IP =100 was defined as “full remission”.

### Procedures

After the subjects were enrolled, they were treated for four weeks with venlafaxine extended-release, one 75 mg capsule per day. Because insomnia is a common complaint in patients with MDD, zolpidem (10 mg per tablet) was prescribed as needed at night only in the first 2 weeks of treatment, with a total amount ≤6 tablets. Four weeks later, the same scales were administered. The HAMD was re-evaluated by the same psychiatrist. Only subjects who complied with venlafaxine extended-release treatment ≥80% (total capsules that the subject took/total days) were included in the post-treatment analyses.

### Statistical methods

All statistical analyses were performed using SPSS for Windows 12.0 (SPSS Inc., Chicago, IL, USA). The independent *t* test, paired *t* test, Pearson’s correlation, and Chi-square test were used in appropriate situations.

Four regression models were used to investigate the impacts of migraine and anxiety disorders on PPS after controlling for the HAMD scores and demographic variables. The first and second models, performed by multiple linear regressions with forward selection, investigated the impacts of migraine and anxiety disorders on pain intensities at baseline and post-treatment, respectively. The third and fourth models, performed by logistic regressions with forward selection, investigated the impacts of migraine and anxiety disorders on treatment response (IP ≥ 50) and full remission (IP = 100) of pain intensities, respectively. The first model investigated the impacts of migraine and anxiety disorders at baseline; therefore, the total subjects at baseline (*N* = 155) were used for analysis. The second to fourth models investigated the impacts of migraine and anxiety disorders post-treatment; therefore, only patients with good compliance (*N* = 85) were used. In the first and second models, the dependent variables were VAS scores (pain intensities of each pain) at baseline and post-treatment, respectively. In the third and fourth models, the dependent variables were treatment response and full remission, respectively. For the four regression models, the independent variables included five demographic variables (age, gender, educational years, marital status, and employment status), migraine, anxiety disorders, and the severity of depression (HAMD scores at baseline for the first, third, and fourth models and post-treatment for the second model). In all statistical analyses, a two-tailed *P* value <0.05 was considered statistically significant.

## Results

### Subjects

During the study period, 155 patients (49 men, 106 women) agreed to participate. Among them, 16 patients had been treated with antidepressants or other psychotropic drugs and 13 and 3 patients had quit these medications for at least three months and six weeks, respectively, before enrollment. Six patients had a history of alcohol abuse and all of them had achieved full remission for at least 3 months.

Table 1 shows their demographic variables. Among 155 patients, 85 (54.8%) subjects with good compliance (venlafaxine treatment ≥80% compliance) were included in the post-treatment analyses (treatment group). The mean amount of zolpidem taken during the first two-week treatment period was 2.1 ± 2.4 tablets. The other 70 subjects (withdrawal group) included 22 subjects who had poor compliance (compliance <80%) or were shifted to other antidepressants and 48 subjects who did not finish the treatment. There were no significant differences at baseline in the five demographic variables, the percentages of migraine and anxiety comorbidities, and the HAMD scores between the treatment and withdrawal groups (Table 1). In analyzing results post-treatment, only the treatment group was used.

**Table 1:** Demographic variables and comorbidities among 155 patients with major depressive disorder

	**Total**	**Treatment group**	**Withdrawal group**
Case number	155	85	70
Age (years)	30.3 ± 8.0	29.5 ± 7.3	31.2 ± 8.7
Educational years	13.4 ± 2.5	13.6 ± 2.7	13.1 ± 2.2
Female (%)	68.4	63.5	74.3
In employment (%)	59.4	56.5	62.9
Married (%)	38.7	38.8	38.6
With migraine (%)	47.1	51.8	41.4
With any anxiety disorder (%)	45.2	50.6	38.6
HAMD scores	23.4 ± 4.0	23.2 ± 3.9	23.6 ± 4.1

*Abbreviation: HAMD* Hamilton Depression Rating scale.

### Headache and psychiatric diagnoses

Among the 155 participants, 73 (47.1 %) had migraine, including 16 patients with chronic migraine (code 1.3), two with chronic migraine with medication overuse headache (codes 1.3 and 8.2), 49 with episodic migraine without aura (code 1.1), and 6 with episodic migraine both with and without aura (code 1.2). The other 82 subjects included 27 (17.4 %) with probable migraine without aura (code 1.5.1), one (0.6 %) with chronic tension-type headache (TTH) (code 2.3), 26 (16.8 %) with frequent episodic TTH (code 2.2), four (2.6 %) with infrequent episodic TTH (code 2.1), six (3.9%) with probable episodic TTH (code 2.4), nine (5.8 %) with headache unspecified (code 14.2), and nine (5.8%) without any headaches.

Among the 155 patients, 70 (45.2%) subjects had at least one anxiety comorbidity, including 9.7% (*N* = 15) with panic disorder and/or agoraphobia, 24.5% (*N* = 38) with social phobia, 18.1% (*N* = 28) with specific phobia, 11.6% (*N* = 18) with post-traumatic stress disorder, 6.5% (*N* = 10) with obsessive-compulsive disorder, and 7.1% (*N* = 11) with GAD.

Among the 85 patients in the treatment group, 44 (51.8%) patients had migraine, including 10 patients with chronic migraine (code 1.3), two with chronic migraine with medication overuse headache (codes 1.3 and 8.2), 27 with episodic migraine without aura (code 1.1), and 5 with episodic migraine both with and without aura (code 1.2), and 43 (50.6%) patients had any one of the anxiety disorders. There were no significant differences in the five demographic variables between those with and without migraine as well as between those with and without anxiety comorbidities.

Migraine and anxiety disorders are also comorbid with each other. Compared with MDD patients without migraine, MDD patients with migraine had a higher risk of comorbidity with any anxiety disorder (63.4% *vs*. 28.0%, odds ratio (OR) = 4.64, *P* <0.001 at baseline; 72.7% *vs*. 26.8%, OR = 7.27, *P* <0.001 post-treatment).

### The percentages of PPS between groups

Table 2 shows the percentages of PPS in the treatment group between groups. Patients with migraine had significantly higher percentages in all PPS at baseline and post-treatment as compared with patients without migraine. Significant differences between patients with and without anxiety disorders were noted in terms of headache at baseline and pains in the neck/shoulder and limbs post-treatment.

**Table 2:** **The percentages of pains at baseline and post-4-week treatment between groups in 85 patients with major depressive disorder**^**a**^

	**Pre-treatment**						**Post-treatment**					
**Groups**	**Migraine**			**Anxiety disorders**			**Migraine**			**Anxiety disorders**		
	**Yes**	**No**	**OR (95% CI)**	**Yes**	**No**	**OR (95% CI)**	**Yes**	**No**	**OR (95% CI)**	**Yes**	**No**	**OR (95% CI)**
Bone and/or Joints	70.5	36.6**	4.1 (1.7-10.2)	55.8	52.4	1.1 (0.5-2.7)	50.0	22.0*	3.6 (1.4-9.2)	46.5	26.2	2.5 (0.98-6.1)
Head	100	68.3**	-	95.3	73.8**	7.3 (1.5-35.2)	88.6	53.7**	6.7 (2.2-20.5)	76.7	66.7	1.7 (0.6-4.3)
Back	81.8	46.3**	5.2 (2.0-13.9)	69.8	59.5	1.6 (0.6-3.8)	63.6	26.8**	4.8 (1.9-12.0)	55.8	35.7	2.3 (0.95-5.4)
Abdomen	70.5	36.6**	4.1 (1.7-10.2)	55.8	52.4	1.1 (0.5-2.7)	50.0	24.4*	3.1 (1.2-7.8)	41.9	33.3	1.4 (0.6-3.5)
Chest	70.5	41.5**	3.4 (1.4-8.3)	60.5	52.4	1.4 (0.6-3.3)	43.2	12.2**	5.5 (1.8-16.6)	30.2	26.2	1.2 (0.5-3.1)
Neck and/or shoulder	97.7	80.5*	10.4 (1.2-87.5)	88.4	90.5	0.8 (0.2-3.2)	90.9	53.7**	8.6 (2.6-28.6)	83.7	61.9*	3.2 (1.1-8.8)
General muscle	79.5	53.7*	3.4 (1.3-8.7)	69.8	64.3	1.3 (0.5-3.2)	56.8	26.8**	3.6 (1.4-8.9)	51.2	33.3	2.1 (0.9-5.0)
Limbs	54.5	24.4**	3.7 (1.5-9.4)	46.5	33.3	1.7 (0.7-4.2)	36.4	14.6*	3.3 (1.2-9.6)	37.2	14.3*	3.6 (1.2-10.3)

*P <0.05; **P <0.01.

*Abbreviations: OR* odds ratio, *CI* confidence interval.

^a^The case numbers of the “migraine” and “anxiety disorders” groups were 44 (51.8%) and 43 (50.6%), respectively.

Regarding the total number of PPS, patients with migraine had significantly higher numbers of PPS at baseline (6.3 ± 1.9 vs. 3.9 ± 2.1, *P* <0.001) and post-treatment (4.8 ± 2.4 vs. 2.3 ± 2.1, *P* <0.001) as compared with patients without migraine. Similarly, patients with anxiety disorders also tended to have a higher number of PPS at baseline (5.4 ± 2.3 vs. 4.8 ± 2.3, *P* = 0.21) and post-treatment (4.2 ± 2.5 vs. 3.0 ± 2.4, *P* = 0.02) as compared with patients without anxiety disorders.

### Differences in the severity of depression and pains between groups

Table 3 shows that the patients with migraine had a significantly greater severity of depression and significantly higher pain intensities and frequencies in all PPS at baseline and post-treatment as compared with the patients without migraine, with the exception of chest pain at baseline.

**Table 3:** **The severities of pain indices and depression at baseline and post-4-week treatment between groups in 85 patients with major depressive disorder**^**a**^

		**Pre-treatment**				**Post-treatment**			
		**Migraine**		**Anxiety disorders**		**Migraine**		**Anxiety disorders**	
		**Yes (*N*** **= 44)**	**No (*N*** **= 41)**	**Yes (*N*** **= 43)**	**No (*N*** **= 42)**	**Yes (*N*** **= 44)**	**No (*N*** **= 41)**	**Yes (*N*** **= 43)**	**No (*N*** **= 4 2)**
HAMD scores		24.2 ± 3.9	22.2 ± 3.5*	24.1 ± 4.1	22.3 ± 3.5*	14.8 ± 6.6	10.6 ± 5.8**	13.4 ± 7.0	12.1 ± 6.0
Bone and/or joints	Intensity	3.7 ± 3.2	1.3 ± 3.1**	3.1 ± 3.3	1.9 ± 2.5	2.1 ± 3.1	0.5 ± 1.0**	1.6 ± 2.7	1.0 ± 2.2
	Frequency	3.1 ± 2.9	1.2 ± 2.1**	2.7 ± 3.0	1.6 ± 2.2	1.7 ± 2.4	0.7 ± 1.7*	1.6 ± 2.4	0.8 ± 1.8
Head	Intensity	6.4 ± 2.6	2.4 ± 2.5**	5.1 ± 3.0	3.8 ± 2.4	4.0 ± 3.2	2.0 ± 2.5**	3.1 ± 3.2	2.9 ± 3.0
	Frequency	4.7 ± 2.4	1.8 ± 2.1**	3.7 ± 2.6	2.8 ± 2.8	3.2 ± 2.4	1.1 ± 1.4**	2.8 ± 2.5	1.6 ± 1.8*
Back	Intensity	4.3 ± 3.0	1.6 ± 2.3**	3.6 ± 3.3	2.5 ± 2.6	2.1 ± 2.7	0.7 ± 1.4**	1.6 ± 2.3	1.2 ± 2.2
	Frequency	3.9 ± 2.8	1.9 ± 2.5**	3.1 ± 2.9	2.7 ± 2.8	2.1 ± 2.4	0.9 ± 2.0*	1.8 ± 2.3	1.2 ± 2.2
Abdomen	Intensity	2.7 ± 2.7	1.2 ± 1.9**	2.2 ± 2.7	1.7 ± 2.2	1.6 ± 2.2	0.5 ± 1.1**	1.3 ± 2.2	0.8 ± 1.4
	Frequency	2.6 ± 2.5	1.0 ± 1.7**	2.3 ± 2.6	1.4 ± 1.9	1.5 ± 2.1	0.5 ± 1.3**	1.5 ± 2.3	0.6 ± 0.9*
Chest	Intensity	3.1 ± 3.4	2.0 ± 2.9	2.7 ± 3.3	2.4 ± 3.2	1.8 ± 2.8	0.3 ± 0.8**	1.2 ± 2.4	0.9 ± 2.1
	Frequency	2.3 ± 2.5	1.5 ± 2.4	1.8 ± 2.3	2.0 ± 2.7	1.1 ± 1.7	0.2 ± 0.6**	0.7 ± 1.4	0.7 ± 1.3
Neck and/or shoulder	Intensity	6.9 ± 2.6	3.7 ± 3.2**	5.9 ± 3.5	4.8 ± 3.0	4.1 ± 3.4	1.6 ± 2.1**	3.4 ± 3.3	2.4 ± 2.8
	Frequency	5.9 ± 2.0	3.8 ± 3.0**	5.1 ± 2.8	4.7 ± 2.7	4.2 ± 2.4	2.4 ± 2.9**	4.0 ± 2.7	2.7 ± 2.8*
General muscle	Intensity	4.4 ± 3.1	1.9 ± 2.6**	3.4 ± 3.0	3.0 ± 3.3	2.5 ± 3.3	0.8 ± 1.7**	1.9 ± 2.9	1.5 ± 2.6
	Frequency	4.0 ± 3.0	2.1 ± 2.6**	3.5 ± 3.1	2.5 ± 2.7	2.3 ± 2.9	1.0 ± 2.2*	2.0 ± 2.9	1.3 ± 2.4
Limbs	Intensity	2.1 ± 2.5	0.8 ± 1.8**	1.7 ± 2.5	1.2 ± 2.2	1.1 ± 2.0	0.3 ± 1.0*	0.9 ± 1.7	0.5 ± 1.5
	Frequency	1.9 ± 2.5	0.8 ± 1.9*	1.8 ± 2.6	1.0 ± 1.8	1.0 ± 1.7	0.4 ± 1.2*	0.9 ± 1.6	0.5 ± 1.4

*P <0.05; **P <0.01.

*Abbreviation: HAMD* Hamilton Depression Rating scale.

^a^Pain intensity and frequency were measured using a 0–10 visual analog scale and number of pain days in the past week, respectively.

In patients both with and without migraine, the severity of depression, the VAS scores and the frequencies of the eight PPS all showed significant (all *P* <0.05) improvement post-treatment, except for the intensities of pains in the head and limbs and the frequencies of pains in bone/joints, head, abdomen, and limbs among patients without migraine.

Comparing patients with and without anxiety disorders, significant differences were noted in only the severity of depression at baseline and the frequencies of pains in the head, abdomen, and neck/shoulder post-treatment.

For patients with and without any anxiety disorders, the HAMD scores, VAS scores, and pain frequencies at baseline showed significant improvement post-treatment, except for the intensities of pains in the head and limbs and the frequency of pain in the limbs among patients without anxiety disorders.

### The correlation of pain intensities and depression

At baseline (*N* = 155), the HAMD score was significantly correlated to all pain intensities, with correlation co-efficiencies ranging from 0.39 (headache) to 0.20 (abdominal pain). Post-treatment (*N* = 85), the correlation co-efficiencies (all *P* <0.05) ranged from 0.35 (pain in bone/joint) to 0.23 (back pain). The correlations of age and educational levels with all pain intensities were not significant at baseline and post-treatment.

### Differences in the percentages of treatment response and full remission between groups

Table 4 shows the trend that patients with migraine had lower percentages of treatment response and full remission in the PPS, except for pain in bone/joints. Statistically significant differences between patients with and without migraine were noted in chest pain for treatment response and in chest pain, headache, and neck/shoulder pain for full remission.

**Table 4:** **The percentages of treatment response and full remission post-4-week treatment between groups in 85 patients with major depressive disorder**^**b,c**^

	**Treatment response**						**Full remission**					
**Groups**	**Migraine**			**Anxiety disorders**			**Migraine**			**Anxiety disorders**		
	**Yes**	**No**	**OR (95% CI)**	**Yes**	**No**	**OR (95% CI)**	**Yes**	**No**	**OR (95% CI)**	**Yes**	**No**	**OR (95% CI)**
Bone and/or joints	67.7	66.7	1.1 (0.3-3.9)	70.8	63.6	1.4 (0.4-4.8)	45.2	60.0	0.5 (0.2-1.9)	45.8	54.5	0.7 (0.2-2.3)
Head	45.5	60.7	0.5 (0.2-1.4)	53.7	48.8	1.2 (0.5-3.1)	11.4	39.3**	0.2 (0.06-0.7)	19.5	25.8	0.7 (0.2-2.1)
Back	69.4	73.7	0.8 (0.2-2.8)	73.3	68.0	1.3 (0.4-4.2)	36.1	63.2^a^	0.3 (0.1-1.0)	40.0	52.0	0.6 (0.2-1.8)
Abdomen	61.3	86.7^a^	0.2 (0.05-1.3)	58.3	81.8	0.3 (0.1-1.2)	38.7	66.7	0.3 (0.1-1.2)	33.3	63.6^a^	0.3 (0.1-0.96)
Chest	48.4	94.1**	0.1 (0.01-0.5)	65.4	63.6	1.1 (0.3-3.5)	41.9	76.5*	0.2 (0.06-0.8)	53.8	54.5	0.97 (0.3-3.0)
Neck and/or shoulder	46.5	60.6	0.6 (0.2-1.4)	47.4	57.9	0.7 (0.3-1.6)	7.0	39.4**	0.1 (0.03-0.5)	10.5	31.6*	0.3 (0.1-0.9)
General muscle	62.9	63.6	0.97 (0.3-2.9)	63.3	63.0	1.0 (0.3-3.0)	37.1	54.5	0.5 (0.2-1.5)	33.3	55.6	0.4 (0.1-1.2)
Limbs	62.5	90.0	0.2 (0.02-1.7)	65.0	78.6	0.5 (0.1-2.4)	41.7	70.0	0.3 (0.06-1.5)	35.0	71.4^a^	0.2 (0.05-0.95)

*P <0.05; **P <0.01.

*Abbreviation: CI* confidence interval.

^a^Borderline significance (*P* =0.1-0.05).

^b^The case numbers of the “migraine” and “anxiety disorders” groups were 44 (51.8%) and 43 (50.6%), respectively.

^c^Treatment response and full remission were defined as an improvement percentage of pain intensities > = 50 and =100, respectively.

Patients with anxiety disorders also tended to have lower percentages of full remission post-treatment, and a significant difference was only noted in neck/ shoulder pain (Table 4).

### The impacts of migraine on PPS at baseline and post-treatment

The first regression model demonstrated that migraine independently predicted the intensities of the eight PPS at baseline, after controlling for the demographic variables and HAMD scores (Table 5). Moreover, the impacts of migraine might be greater than the severity of depression, except for pains in the chest and limbs. The second regression model demonstrated that migraine independently predicted the intensities of pains in bone/joints, back, chest, and neck/shoulder post-treatment. In the two regression models, anxiety disorders were not entered.

**Table 5:** **Independent factors predicting the intensities of pains at baseline and post-4-week treatment**^**a,b,c**^

**Locations**	**Time points**	**Independent variable**	**Beta**	***R***^***2***^**change**	***t***	***P***
Bone and/or joints	**Baseline**	**Migraine**	**0.32**	**0.16**	**4.26**	**< 0.01**
		**HAMD**	**0.28**	**0.07**	**3.83**	**< 0.01**
	*Post Tx*	*HAMD*	*0.25*	*0.12*	*2.38*	*0.02*
		*Migraine*	*0.27*	*0.06*	*2.54*	*0.01*
		*Job*	*-0.20*	*0.04*	*-2.01*	*0.048*
Head	**Baseline**	**Migraine**	**0.46**	**0.29**	**6.70**	**< 0.01**
		**HAMD**	**0.25**	**0.06**	**3.68**	**< 0.01**
	*Post Tx*	*HAMD*	*0.58*	*0.33*	*6.33*	*< 0.01*
Back	**Baseline**	**Migraine**	**0.17**	**0.36**	**4.75**	**< 0.01**
		**HAMD**	**0.03**	**0.19**	**2.48**	**0.01**
	*Post Tx*	*HAMD*	*0.42*	*0.24*	*4.54*	*< 0.01*
		*Age*	*0.37*	*0.09*	*4.05*	*< 0.01*
		*Migraine*	*0.22*	*0.04*	*2.36*	*0.01*
		*Job*	*-0.21*	*0.04*	*-2.24*	*0.03*
Abdomen	**Baseline**	**Migraine**	**0.28**	**0.08**	**3.63**	**< 0.01**
	*Post Tx*	*HAMD*	*0.34*	*0.12*	*3.34*	*< 0.01*
		*Age*	*0.20*	*0.04*	*1.99*	*0.05*
Chest	**Baseline**	**HAMD**	**0.32**	**0.13**	**4.06**	**< 0.01**
		**Migraine**	**0.16**	**0.01**	**2.00**	**0.048**
	*Post Tx*	*HAMD*	*0.38*	*0.20*	*3.74*	*< 0.01*
		*Migraine*	*0.23*	*0.05*	*2.26*	*0.03*
Neck and/or shoulder	**Baseline**	**Migraine**	**0.39**	**0.15**	**5.25**	**< 0.01**
	*Post Tx*	*HAMD*	*0.46*	*0.30*	*5.02*	*< 0.01*
		*Migraine*	*0.27*	*0.06*	*2.92*	*< 0.01*
		*Educational Year*	*-0.18*	*0.03*	*-2.03*	*0.046*
General muscle	**Baseline**	**Migraine**	**0.32**	**0.16**	**4.23**	**< 0.01**
		**HAMD**	**0.28**	**0.07**	**3.69**	**< 0.01**
	*Post Tx*	*HAMD*	*0.27*	*0.51*	*5.59*	*< 0.01*
		*Age*	*0.04*	*0.20*	*2.17*	*0.03*
Limbs	**Baseline**	**HAMD**	**0.26**	**0.11**	**3.43**	**< 0.01**
		**Migraine**	**0.22**	**0.04**	**2.87**	**< 0.01**
		**Job**	**-0.19**	**0.04**	**-2.54**	**0.01**
	*Post Tx*	*Age*	*0.36*	*0.08*	*3.36*	*< 0.01*
		*HAMD*	*0.26*	*0.08*	*2.65*	*0.01*
		*Job*	*-0.22*	*0.04*	*-2.06*	*0.04*

*Abbreviations: HAMD* Hamilton Depression Rating scale, post Tx post-four-week treatment.

^a^Multiple linear regressions were used in this Table.

^b^The case numbers included in the analyses at baseline and post-treatment were 155 and 85, respectively.

^c^Data in boldface indicate data at baseline.

### Factors independently predicting the treatment response and full remission

The third and fourth (logistic) regression models demonstrated that migraine independently predicted the treatment response for chest pain (OR = 0.06, 95% CI = 0.01-0.50, *P* = 0.01) and full remission for pains in the head (OR = 0.15, 95% CI = 0.04-0.56, *P* = 0.01), chest (OR = 0.22, 95% CI = 0.06-0.84, *P* = 0.03), and neck/shoulder (OR = 0.12, 95% CI = 0.03-0.45, *P* <0.01) after controlling for the demographic variables and HAMD score at baseline. Anxiety disorders independently predicted full remission for pains in the abdomen (OR = 0.29, 95% CI = 0.09-0.96, *P* = 0.04) and limbs (OR = 0.22, 95% CI = 0.05-0.95, *P* = 0.04).

## Discussion

Compared with patients without migraine, patients with migraine had a significantly higher number of PPS and higher pain intensities and frequencies in all PPS at baseline and post-treatment, with the exception of chest pain at baseline. After controlling for demographic variables, depression, and anxiety comorbidities, migraine independently predicted all PPS at baseline and four pains post-treatment (Table 5). These results implied two issues. 1) Migraine is an important factor related to PPS among patients with MDD. Although the severity of depression was significantly correlated to pain intensities, it could not explain all pain intensities. 2) The impacts of migraine on PPS might be greater than those of anxiety disorders among patients with MDD. However, this might need more evidence. Migraine and anxiety disorders are often comorbid with each other [29,30]. Anxiety disorders are also comorbid with PPS [19-21]. Therefore, investigation of PPS among depressive and anxiety disorders should consider the impacts of migraine.

The result that the impacts of migraine on most PPS at baseline were greater than the severity of depression might be explained the following hypothesis. At baseline, subjects with migraine all suffered from headache (Table 2) and had a high headache intensity (VAS = 6.4 ± 2.6) and frequency (4.7 ± 2.4 days) in the past week (Table 3). Migraine is often accompanied with signs of increased intracranial and extracranial mechanical sensitivities and related to somatosensory amplification [31,32]. Repeated migraine attacks might cause an effect similar to central sensitization, which is related to allodynia, hyperalgesia and spontaneous pain and might allow subjects to become more sensitive to other PPS [22,31]. Moreover, headache intensity is related to muscloskeletal symptoms [33]. Post-treatment, the headache intensity and frequency of migraine were decreased. The effect of central sensitization resulting from headache might be decreased. Therefore, the impact of depression (HAMD scores post-treatment) on PPS might exceed the impact of migraine. In future studies, the impacts of headache attacks on other PPS might need to be further clarified. There is a possibility that treatment of migraine might help to improve headache attacks, then improve other PPS. The patients with migraine had significantly higher HAMD scores at baseline and post-treatment than the patients without migraine (Table 3). A trend was noted that the patients with migraine had a smaller reduction in the HAMD score post-treatment (9.4 points *vs*. 11.6 points for the migraine group vs. non-migraine group) as compared with the patients without migraine, and the difference in the HAMD scores between the migraine and non-migraine groups became greater (2.0 points at baseline and 4.2 points post-treatment) (Table 3). These results implied the possibility that migraine might be a factor related to a poorer prognosis of depression. However, more evidence is still needed. Moreover, the impacts of anxiety disorders should also be further studied.

After controlling for demographic variables and depression at baseline, migraine independently predicted a lower percentage of treatment response in chest pain and of full remission in pains in the chest, head, and neck/shoulder. This demonstrated that migraine also has negative impacts on the treatment prognosis of PPS. Patients with noncardiac chest pain are often comorbid with depression and anxiety disorders [19,20]. Patients with migraine have a higher risk of chest pain, and a link between migraine and vasospastic disorders might be one of the possible reasons [34]. The possible reasons for which migraine has a negative impact on the recovery of chest pain should be investigated in future studies. Migraine was also an independent factor predicting a poor treatment prognosis of neck/shoulder pain. Headache is a strong factor associated with neck/shoulder soreness and musculoskeletal symptoms [33]. Moreover, patients with migraine have muscle hyperalgesia and decreased pressure pain thresholds in the neck/shoulder [35]. These might be the possible reasons for which migraine has a negative impact on the recovery of neck/shoulder pain post-treatment. Comorbidity with anxiety disorders was an independent factor related to poor treatment remission of abdominal pain. Recurrent abdominal pain is associated with anxiety disorders [36]. In fact, patients with migraine also have a higher risk of abdominal pain [37]. The interaction of abdominal pain, anxiety disorders, and migraine might need to be clarified.

Several methodological issues or limitations need to be addressed. 1) The analyses post-treatment only included patients with good compliance. Only 85 (54.8%) subjects with good compliance were included in the post-treatment analyses. Although there were no significant differences in the demographic variables and the HAMD scores between the treatment and withdrawal groups, possible bias was unable to be fully excluded, because patients with a poor treatment response might be more likely to drop out [38]. 2) This study had no placebo-controlled group because the major purpose was not to investigate the efficacy of venlafaxine. Therefore, the improvement of PPS and depression might be mixed with multiple effects. 3) The headache parameters in the past week were recalled by the patients. Collecting data prospectively using a headache diary might be more reliable. However, withholding pharmacological treatment while prospectively observing headache parameters might result in ethical problems owing to the suicidal risk. 4) This study only observed the four-week treatment response, because four-week treatment may be a milestone after which a treatment response can be observed [39]. 5) Although the total mean amount of zolpidem taken in the first two weeks was only 2.1 ± 2.4 tablets, possible bias resulting from zolpidem usage could not be excluded. 6) One previous study demonstrated that MDD patients with chronic migraine had the worst bodily pain on the SF-36, followed by episodic migraine [40]. Therefore, chronic migraine might have a greater contribution to influence PPS and treatment response than episodic migraine. In future study, a larger sample size should be used to compare the impacts of chronic migraine, episodic migraine, TTH, and other headache types on PPS and treatment response.

## Conclusion

After controlling for depression, anxiety comorbidities, and demographic variables, migraine independently predicted the eight PPS at baseline and four PPS post-treatment. Moreover, migraine also independently predicted poor treatment prognosis of pains in the chest, head, and neck/shoulder. Anxiety disorders predicted poor treatment prognosis of pains in the abdomen and limbs. Depression, anxiety, migraine, and PPS might interact with each other. The impacts of migraine and anxiety disorders should be considered in investigating PPS among patients with MDD. Integration of the treatment for migraine and anxiety disorders into the treatment of depression might help improve PPS and the prognosis of MDD.

## Abbreviations

GAD: Generalized anxiety disorder; HAMD: Hamilton Depression Rating Scale; IP: Improvement percentage; ICHD-2: International Classification of Headache Disorders 2nd edition; MDD: Major depressive disorder; OR: Odds ratio; PPS: Painful physical symptoms; TTH: Tension-type headache; VAS: visual analog scale.

## Competing interests

The authors declare that they have no competing interests.

## Authors’ contributions

SJW and CIH designed the study and wrote the protocol. CIH, CYL, CYC, and CHY collected the data. CIH and SJW undertook the statistical analysis, and CIH wrote the first draft of the manuscript. All authors contributed to and have approved the final manuscript.
